# Supplementary material for: Barriers and facilitators to parents’ engagement with and perceived impact of a childhood obesity app: A mixed-methods study
Source: PLOS Digit Health. 2024 Mar 27;3(3):e0000481. doi: 10.1371/journal.pdig.0000481 (PMC10971669; doi:10.1371/journal.pdig.0000481)
Supplement: S1 Text — (DOCX) [file pdig.0000481.s001.docx]

Semi-structured interview guide

[Introduce interviewer]

Before we start, is it okay with you if I audio-record this session?

- The reason we record is so we can go back and remember what was said and what wasn’t said. Destroyed as soon as study is done, any publications don’t use names
  - *Ask again for confirmation once recording is on*
  - If no: “That’s fine, is it okay if I type notes as we talk and share those notes with you once we have finished the interview so you can make sure they are accurate?”

Purpose of our investigation is to evaluate the app - we weren’t involved in development, we just want to figure out what is good and can proceed and what needs to improve (any positive / negative feedback is useful). Your feedback is very valuable for us to evaluate the app.

Today we’re going to be going through some questions to discuss your experiences using the app. There are six main aspects about the app that you will be asked about, and then you will have the opportunity to provide feedback on anything else you want to talk about that you feel we haven’t covered. If there is anything you don’t understand during the interview, feel free to ask!

Before we get into those questions, I just wanted to remind you that all of your answers will be kept confidential and stored on a password-protected computer that only the research team can access. The audio recording that we’re taking today will be deleted as soon as it has been transcribed, and any identifying information (like your name) will be removed from the audio recording before it is sent to the transcription service.

So before we get started, do you have any questions?

**Indented questions were used as prompts to encourage the participant to provide more detail, when necessary*

**1) General usability**

- How would you describe your and your family’s experience using the ‘NoObesity’ app?
  - **How much did you like or dislike using the app? Why?*
  - *How easy or difficult did you find the app to use? Why?*
- What has happened as a result of using the app with your family?
  - *Has your behaviour changed? If so, how?*
  - *Can you tell me a bit more about how the app helped you and your family support that change?*

**2) Self-monitoring and goal-setting**

- What do you think of the app function that helps to monitor your family’s habits (the ‘Record Family Progress’ section)?
- Did the app help you set goals? If so, how come?

**3) Physical activity and healthy eating support**

- Did the app support your family to be more active? If so, how?
- Did the app support your family to eat healthy? If so, how?

**4) Weight and health assessment**

- What did you think about the app’s weight and health measurements (the ‘Family Survey’ section)?
  - *Did you use it? Did you like or dislike it? Why?*

**5) Personalised feedback and motivational strategies**

- What did you think of the app’s feedback?
  - *This can include any information you were provided by the app in response to something you did on the app*
  - *For example, feedback from the app on health information that you added to the family survey section / getting points in challenges*
- Did the app motivate your family?
  - *What helped or didn’t help?*
  - *How come?*

**6) Social support and health care expert involvement**

- Did you share and collaborate with any others who were also using the app? Eg. any HCPs?
  - *If yes, how?*
    - *What did you like or dislike about that and why?*
  - *If no, why not?*
- Was your health care worker involved in using the app? If so, can you explain how?
  - *Hypothetical (if possible, would that be something you’d use? What would be useful about that for you or a HCP you’re working with?)*
  - **Context**: app was developed as a facilitation tool because HEE found it was hard for HCPs to speak with families about weight, and they didn’t have good training/tools to talk about in the context of family and children, felt uncomfortable because sensitive topic
    - *What are your reflections on that?*
    - *How would you respond to getting feedback from / interacting with doctor through app?*

**7) Feedback**

- We found there were a lot of strong views (positive and negative) about the name of the app in early feedback during recruitment, what is your view?
  - *Name of the app came from a competition HEE had with children*
- What would you suggest to make the app better?
  - *Can you tell me more about that, and how you think it would improve the app?*
  - *Are there any other suggestions that you think would make the app better?*
- Do you plan to continue using the app after the completion of the study?
- Is there anything else you’d like to mention?

If you have any questions or comments that you want to add after the interview is over, you are welcome to email me or another researcher on the information sheet.

What next:

- Three months from now - send a survey via email, just to get further feedback on your thoughts and experiences
- At the conclusion at that point, we will be analysing feedback from these sessions and surveys to provide recommendations to HEE because they plan to create a new version of the app
- We will publish data in academic journals, but all data will be anonymous
- Also creating a website and public materials, which will be sent to everyone to overview
- Amazon voucher (100 pounds) - everyone who completes study

Do you have any questions?

Demographic Questionnaire

1. AGE: In what year were you born?
   1. (If refuse to answer): Would you mind indicating which of these age bands you are in: 18-24, 25-34, 35-44, 45-54, 55-64, 65-74, 75-84, 85+
2. GENDER: How do you currently describe your gender identity?
3. FAMILY:
   1. Are you…? Single, Married, Living together with a partner, Divorced/separated, Widowed
   2. How many adults live in your household (people age 18 or more)? [WRITE IN]
   3. How many children (people age 17 or less) live in your household? [WRITE IN]
4. ETHNICITY: To which of these groups do you consider you belong?
   1. ASIAN: of Indian origin
   2. ASIAN: of Pakistani origin
   3. ASIAN: of Bangladeshi origin
   4. ASIAN: of Chinese origin
   5. ASIAN: of any other origin (WRITE IN) ___________________________
   6. BLACK: of African origin
   7. BLACK: of Caribbean origin
   8. BLACK: of other origin (WRITE IN) ___________________________
   9. WHITE: of British origin
   10. WHITE: of Scottish origin
   11. WHITE: of Welsh origin
   12. WHITE: of any other origin (WRITE IN) ___________________________
   13. OTHER (WRITE IN)
   14. Don’t know
5. URBAN: Would you describe the place where you live as a big city, the suburbs or outskirts of a big city, a small city or town, a country village, or, a farm or home in the country?
   1. A big city
   2. The suburbs or outskirts of a big city
   3. A small city or town
   4. A country village
   5. A farm or home in the country
   6. Other
   7. Don’t know
6. INCOME: The incomes of households differ a lot in Britain today. Here is a table showing the range of incomes that people have. Which option best represents the total income of your household before tax? Please select one answer only.
   1. Up to £12.500 per year
   2. £12.500 up to £20.000 per year
   3. £20.000 up to £30.000 per year
   4. £30.000 up to £40.000 per year
   5. £40.000 up to £50.000 per year
   6. £50.000 up to £60.000 per year
   7. £60.000 up to £70.000 per year
   8. £70.000 up to £80.000 per year
   9. Over £80.000 per year
   10. Prefer not to say
7. EDUCATION: What is the highest educational or vocational qualification that you have or that you will receive if you complete your next set of exams?
   1. No qualifications
   2. 5 or more GCSE grades A-C
   3. 4 or less GCSE grade A-C
   4. GCSE grade D-G
   5. 5 or more Scottish Standard Grades, grades 1-3
   6. 4 or less Scottish Standard Grades, grades 1-3
   7. 5 or more O Grades, grades 1-3 (Scottish Ordinary Grades)
   8. 4 or less O Grades, grades 1-3 (Scottish Ordinary Grades)
   9. Scottish Standard Grades, grades 4-7
   10. Scottish Highers (either SCE or SQC)
   11. Scottish Certificate Sixth Year Studies
   12. SVQ level 1 or 2 (Scottish Vocational Qualifications)
   13. SVQ level 3 ( “ )
   14. SVQ level 4 ( “ )
   15. SVQ level 5 ( “ )
   16. CSEs
   17. 5 or more O levels
   18. 4 or less O levels
   19. GCE A levels or equivalent
   20. NVQ level 1 or 2
   21. NVQ level 3 or 4
   22. NVQ level 5
   23. GNVQ Foundation
   24. GNVQ Intermediate
   25. GNVQ Advanced
   26. Certificate or Diploma of Higher Education
   27. HND (Higher National Diploma)
   28. Bachelor’s degree
   29. Graduate Certificates and Diploma
   30. Post-degree professional qualification (eg banking accountancy, architecture, etc.)
   31. Master’s Degree
   32. Doctoral Degree
   33. Don’t know
8. EMPLOYMENT: Which of these descriptions best describes your current situation?
   1. Working Full time (30 hours a week or more)
   2. Working Part time (8-29 hours a week)
      1. If (a) or (b): Apart from working, do you also study? (yes / no / DK)
   3. Retired
   4. Unemployed
   5. Permanently sick or disabled
   6. In community or military service
   7. Undergraduate Student
   8. Post graduate student
   9. In full time education (not higher degree)
   10. In part time education (not higher degree)
       1. If (g)-(j): And apart from studying, do you also work? (yes / no / DK)
   11. Doing housework, looking after children or other persons

Final survey

**System Usability Scale (SUS)**

The 10 SUS statements below were presented twice, using a 5-point Likert scale (1=strongly disagree and 5=strongly agree) with the following instructions: (1) “Please rate your agreement with the following statements from your experience using the NoObesity app” and (2) “Please rate the following statements from your child(ren)'s perspective of using the NoObesity app.”

1. I think that I would like to use this system frequently
2. I found the system unnecessarily complex
3. I thought the system was easy to use
4. I think that I would need the support of a technical person to be able to use this system
5. I found the various functions in this system were well integrated
6. I thought there was too much inconsistency in this system
7. I would imagine that most people would learn to use this system very quickly
8. I found the system very cumbersome to use
9. I felt very confident using the system
10. I needed to learn a lot of things before I could get going with this system

**Impact of app**

**Measured with a 5 point Likert scale*

1. Did the NoObesity app help you set goals?
   1. *Strongly disagree - strongly agree
2. Did you achieve your goals?
   1. *Strongly disagree - strongly agree
3. What did you think about the monitoring function (Record Family Progress)?
   1. *Not at all useful - extremely useful
4. Did the NoObesity app support your family to be more active?
   1. *Strongly disagree - strongly agree
5. Did the NoObesity app support your family to eat healthier?
   1. *Strongly disagree - strongly agree
6. Please think about up to 3 goals you set on NoObesity and rate for each how confident you are that you can do those activities as of now. If you did not set 3 goals, please select 'Not Applicable' for Goals 2 and/or 3. Rate your degree of confidence by recording a number from 0 to 100 on the scale below (0=cannot do at all, 50=moderately certain can do, 100=highly certain can do). (*Self-efficacy*)
7. Did you set up the initial family profile (Family Survey section)?
   1. Yes / no
8. If yes, how frequently did you go back to look at the Family Survey section?
   1. Never / once or twice during study / 1-4 times a month / 2-6 times a week / daily
9. What did you think about the HC points / trophies?
   1. *Dislike a great deal - like a great deal
10. What did you think of the information the app provided as feedback?
    1. *Not at all useful - extremely useful
11. How effective was the NoObesity app at motivating your family to be more active?
    1. *Not at all effective - extremely effective
12. How effective was the NoObesity app at motivating your family to eat healthier?
    1. *Not at all effective - extremely effective
13. Please rate the following features on how effective they were at motivating your family (*Not at all effective - extremely effective):
    1. goal-setting
    2. Notifications
    3. Monitoring behaviour (tracking progress)
    4. Points / trophies
    5. Family photo
    6. Feedback from Family Survey about current behaviour
    7. Games
    8. Suggestions for healthier eating
    9. Suggestions of activities
    10. Linking to a healthcare worker
    11. Doing something together as a family
    12. Other (please specify)
14. How likely would you be to link this app with your GP or another healthcare provider?
    1. *Extremely unlikely - extremely likely
15. How comfortable would you be interacting with a doctor on this app?
    1. *Extremely uncomfortable - extremely comfortable
16. How useful do you think it would be to interact with a doctor on this app?
    1. *Not at all useful - extremely useful

**Bandura - self-efficacy questions**

1. Please briefly list any barriers to your use of the app.
2. Please briefly list any barriers to your efforts to meet your goal(s).

**RE-AIM related questions**

1. What did you think about the name of the app (“NoObesity”)?
   1. *Dislike a great deal - like a great deal
2. If you were seeing NoObesity in the app store for the first time, how likely would you be to download it?
   1. *Extremely unlikely - extremely likely
3. Are you still using the NoObesity app?
   1. Yes / no
4. If no, how long (approximately) did you use the NoObesity app for before stopping?
5. If no, why did you stop using the app?

**General feedback**

1. Please briefly list any suggestions you have to make the app better:
2. Do you have any other feedback about the app you want to share?
