# Supplementary material for: Barriers and facilitators to parents’ engagement with and perceived impact of a childhood obesity app: A mixed-methods study
Source: PLOS Digit Health. 2024 Mar 27;3(3):e0000481. doi: 10.1371/journal.pdig.0000481 (PMC10971669; doi:10.1371/journal.pdig.0000481)
Supplement: S3 Table — (DOCX) [file pdig.0000481.s004.docx]

S3 Table developed from the study protocol [1], with BCT column added**^a^**

| **App function** | **Purpose** | **Outcome** | **Family app?** | **Prof. app?** | **BCTs^a^** |
| --- | --- | --- | --- | --- | --- |
| Set family goals | This function enables families to set behavioral goals to support them in their health and wellbeing. This function takes them through a Specific, Measurable, Action-oriented, Realistic, Timed, Evaluated, Reviewed (SMARTER) process to make plans and set goals. | Families set goals using a SMARTER process that is part of the Making Every Contact Count (MECC) program. | Y | N | 1.1, 1.2, 1.4, 1.6 |
| Record family progress | This function allows families to record how they have been progressing with family goals and enables them to evaluate and review their goals if they have not progressed as expected. | Families are able to record their progress and review their goals. | Y | N | 2.3 |
| Update photo | This function is in place for families to be able to upload pictures of themselves doing healthy things. This could be a meal they have cooked, pictures of them on a health walk, etc. | This is used as an engagement tool allowing families to upload photos of things they have done as a way of encouraging them to come back to the app. | Y | N | - |
| Family survey | This function supports families to take stock of where they are around a number of health-related areas (water, fruit, vegetables, physical activity, sleep, screen time, toothbrushing, happiness, body size). These questions can be completed for all members of the family. Once the survey is completed, the app will prompt families to think about setting a goal around one of the areas of the health survey (if they have scored low on it). | It enables collection of baseline data to allow for comparison of change. | Y | N | 1.6, 2.2 |
| Parent’s survival guide | This function supports families in thinking about how they can overcome the challenges that they face when supporting their children around healthy eating, etc. | Families gain solutions on how they could approach issues and challenges using a MECC approach. | Y | N | 1.2 |
| Healthy choices & healthy challenges | These two functions are developed in the form of games within the app that HCPs can play as an approach to learning in a fun way about what makes a healthy diet and what level of physical activity is required to burn off calories. | It enables families to learn about the Eatwell Plate and about physical activity and provides HCPs with knowledge to support families. | Y | Y | 4.1 |
| Useful links | This function provides links to websites for HCPs to access for furthering their own learning and Continuing Professional Development. | It enables families and HCPs to learn about resources available; enables HCPs to be better informed on possible signposting. | Y | Y | - |
| HC Points / Certification | This function encourages families and HCPs to do all of the above as they gain points and awards on further exploring the content of the app. | Families and HCPs have used the app to develop their knowledge and skills in supporting the prevention and management of childhood obesity. | Y | Y | 10.3 |
| Link accounts | This function enables families and HCPs to connect their accounts so that the HCP can see the families’ data and progress. | HCPs will have a better understanding of the families’ behaviours and progress, enabling them to provide more tailored advice in appointments. | Y | Y | 3.1 |
| How to help families | This function enables the HCP to follow a family (through a story), where the family comes into contact with a number of different health care workers. ‎The app then provides the HCP with information on how interactions with the family can be improved for their own practice. | HCPs will have an increased awareness of the potential opportunities that could be missed in supporting families around health and wellbeing.  HCPs will be enabled to reflect on their own practice when coming into contact with families.  HCPs will see (read) how a MECC approach can be used for their own practice in supporting families. | N | Y | 4.1, 6.1 |
| Common issues | This function provides some of the common issues HCPs face when supporting families around healthy eating, diet, and activity. This function offers HCPs with some solutions on how they can overcome the common issues and challenges they face.  The solutions are developed with MECC principles, which allow HCPs to use MECC skills in supporting families. | HCPs are able to learn about solutions they can use to overcome issues and challenges and are enabled to practise these MECC skills. | N | Y | 4.1 |

^a^No specific behaviour change theory was used in the design of the app, the BCTs have been identified here *post hoc* by the authors from the BCT Taxonomy v1 to aid future analyses and comparisons of interventions

**S3 Table References**

1. Meinert E, Rahman E, Potter A, Lawrence W, Van Velthoven M. Acceptability and Usability of the Mobile Digital Health App NoObesity for Families and Health Care Professionals: Protocol for a Feasibility Study. JMIR Res Protoc. 2020;9: e18068. doi:10.2196/18068
