# Supplementary material for: Barriers and facilitators to parents’ engagement with and perceived impact of a childhood obesity app: A mixed-methods study
Source: PLOS Digit Health. 2024 Mar 27;3(3):e0000481. doi: 10.1371/journal.pdig.0000481 (PMC10971669; doi:10.1371/journal.pdig.0000481)
Supplement: S4 Table — (DOCX) [file pdig.0000481.s005.docx]

| **Theme** | **Subtheme** | **Subtheme 2** | **Codes** |
| --- | --- | --- | --- |
| Facilitators / positive feedback | Motivating / engaging | App was motivating | App helped motivate  Accountability  App increased awareness of behaviour  App increased awareness of weight  Liked recording progress?  Recording progress was motivating?  Prompted behaviours |
|  |  | App was engaging | Engagement / use of app?  Engaging kids?  Interactivity?  Kids used the app  Adoption into daily routine |
|  | Perceived behavioural benefits | - | Ate more healthy  More active / outside more  Increased water consumption  Tried new things |
|  | Suggestions were helpful | - | App supported self-efficacy?  Overcoming barriers helps with self-efficacy  Suggestions supported self-efficacy  Liked activity suggestions?  Liked healthy eating swaps?  Liked parent survival guide?  Links to other resources were useful  Goals helped with planning  Liked goal suggestions / found helpful  Guidance (1 - goal setting) |
|  | Good usability / acceptability | - | Easy to use (1 - goal setting)  User friendly / easy to use  Kid friendly  Aesthetic |
|  | Liked features | Liked family aspect | Liked family involvement / doing together |
|  |  | Liked games | Kids liked games?  Participant like games?  Educational |
|  |  | Liked goal setting | Flexibility  Goals helped with planning  Liked goal setting?  Simple goals  Thinking about barriers was useful |
|  |  | Liked app feedback | Notifications useful / lacking?  Feedback useful?  Liked points trophies? |
|  |  | Elements liked in Family Survey | Personalisation (1 - family survey)  Inclusion of other healthy behaviours  Liked oral health addition |
|  |  | Liked NoObesity name | Medical name is good  Opinion of name?  Prevent from downloading? |
|  | Positive opinions about HCP link | - | Accountability  Comfort discussing with HCP?  Communicating via app  Could integrate with other health services / family  HCP feedback useful?  Want human connection through app  Would link with HCP? |
| Barriers / negative feedback | Not motivating / engaging | General | App helped motivate  Engagement / use of app  Recording progress was motivating |
|  |  | Not enough variety - repetitive | Repetitive (games)  Repetitive (goal setting)  Simple goals |
|  |  | Not enough variety - limited content | Limited things to do  *Liked recording progress*  Boring  Nothing new  Mostly used to record progress  Not realistic (healthy swaps)  Looked for suggestions / ideas outside of app  Liked healthy eating swaps  Flexibility (0 - goal setting) |
|  |  | Not memorable | *Liked recording progress*  Not memorable (family survey)  Forgot to keep up |
|  |  | Not engaging for kids | Interactivity  Engaging kids  Kids used the app |
|  |  | Feedback was lacking | Feedback useful  Lack of feedback  Liked points / rewards  Notifications useful/lacking? |
|  |  | Issues with notifications | Daily notifications too much  Didn't take much notice of notifications  Noise of notification frustrating  Notifications came at bad times |
|  | No perceived benefits from app | Didn't improve health behaviour | Didn't help  Already doing behaviour  More active / outside more  Ate more healthy  Healthy eating harder than activity |
|  |  | Didn't support self-efficacy | App supported self-efficacy  Can't overcome some barriers |
|  | Poor usability / acceptability | Poor usability | User friendly / easy to use  Kid friendly  Differences within family |
|  |  | Poor acceptability | Kids liked games  Participants liked games  Liked goal setting  Aesthetic  Poor acceptability |
|  |  | Lack of clarity | Points not clear / need more meaning  Unclear how it would work  Confused about who it was for |
|  |  | Lack of guidance | Lack of guidance (usability)  Easy to use (0 - goal setting)  Guidance (0 - goal setting)  No help function |
|  |  | Specific complaints | Specific complaints  Technical difficulties  Specific complaints (games)  Can't change inputted data  Inaccuracies |
|  | Barriers | Barriers to app use | *Child's age*  *Covid-19*  Device |
|  |  | Barriers to behaviour | Accessibility  *Child's age*  *Covid-19*  Habits / routines  Lack of accountability  Lack of motivation (convenience)  Lack of skills  Lack of time  Obesity management different for kids vs adults  Other health conditions  Picky eaters  Sibling teasing  Weather |
|  | Sensitive topic | Issues with name | Can see why others wouldn't like  Make name more positive  Negative connotations  Opinion of name  Prevent from downloading |
|  |  | Weight / health measurements | Discomfort tracking kids weight  Discomfort tracking own weight |
|  |  | Concerns with linking with HCP | Comfort discussing with HCP  Sensitive topic  App data didn't reflect true behaviour  Felt that it was inappropriate  Would link with HCP  Communicating via app  HCP feedback useful?  Privacy / security concerns  Wouldn't want to bother HCP  Would link with HCP  Depends on clinician |
| Suggestions for improvement | More content and more variety of content | - | More variety  More information / education  Home workouts  Local activities  Ways to overcome personal barriers  Recipes / meal planning / shopping support  Vegetarian / vegan options |
|  | More engagement for kids | - | More family / kid engagement  Competition would motivate kids  Points / rewards could be motivating (esp for kids) |
|  | Improve self-monitoring feature | - | Trends (incl. retrospective review and recording, visualise progress)  Simplify recording progress  Add notes |
|  | Improve notifications | - | Prompts / reminders  Targeted notifications |
|  | Improve usability | - | Interface redesign  Date of birth rather than age  Back button functionality  Intro / guide to use |
|  | Make app more positive | - | Positive feedback / encouragement  Focus on feelings not measurements |
|  | Increased personalisation | - | Personalisation  Separate goals |
|  | Integration | - | Could integrate with other health services / family  Integration with other health devices  Include / promote in schools |
| Misc. | RE-AIM (maintenance) |  | Continue use after study? |
