# Supplementary material for: Barriers and facilitators to parents’ engagement with and perceived impact of a childhood obesity app: A mixed-methods study
Source: PLOS Digit Health. 2024 Mar 27;3(3):e0000481. doi: 10.1371/journal.pdig.0000481 (PMC10971669; doi:10.1371/journal.pdig.0000481)
Supplement: S5 Table — (DOCX) [file pdig.0000481.s006.docx]

| **Theme** | **Subtheme** | **Codes** | **Factor summary** | **Short description of factor** | **Factor association with theme** | **COM-B component** |
| --- | --- | --- | --- | --- | --- | --- |
| Engagement - motivation | Poor acceptability | Kids liked games (0) Participants liked games (0) Liked goal setting (0,1) Aesthetic (0) Poor acceptability | Aesthetic appeal | Visual interface and colour scheme could affect participants' feelings about the app | Positive | Motivation (automatic) |
| Engagement - motivation | Weight / health measurements | Discomfort tracking kids weight Discomfort tracking own weight | Discomfort tracking weight | Parents did not want an emphasis on weight and measurements, particularly for their children | Negative | Motivation (automatic) Motivation (reflective) |
| Engagement - motivation | Feedback was lacking | Feedback useful (0) Lack of feedback Liked points / rewards (0) Notifications useful/lacking? (0) | Feedback / visualisations | Participants all valued feedback from the app about their progress and how their current behaviour compared to healthy recommendations, but many found the app's feedback lack. | Positive | Capability (psychological) Motivation (automatic) |
| Engagement - motivation | Improve self-monitoring feature | Trends (incl. retrospective review and recording, visualise progress) Simplify recording progress Add notes |  |  |  |  |
| Engagement - motivation | Integration | Could integrate with other health services / family Integration with other health devices Include / promote in schools | Integration | A couple participants suggested that enabling the app to connect with wearables or linking it with existing health service delivery could be helpful for engagement and impact | Positive | Opportunity (physical) |
| Engagement - motivation | Liked games | Kids liked games? (1) Participant like games? (1) Educational | Interactivity / gamification | Participants generally liked the idea of having gamification in the app, but many found the current games repetitive and the points/rewards system confusing. Including friendly competition, rewards, and games was a common suggestion to improve children's engagement | Positive | Motivation (automatic) |
| Engagement - motivation | More engagement for kids | More family / kid engagement Competition would motivate kids Points / rewards could be motivating (esp for kids) |  |  |  |  |
| Engagement - motivation | App was engaging | Engagement / use of app? (1) Engaging kids? (1) Interactivity? (1) Kids used the app (2) Adoption into daily routine | *Descriptive sub-theme (not a factor)* | - | - | - |
| Engagement - motivation | Not engaging for kids | Interactivity (0) Engaging kids (0) Kids used the app (0,1) | *Descriptive sub-theme (not a factor)* | - | - | - |
| Engagement - motivation | Continue use after study? | - | *Descriptive sub-theme (not a factor)* | - | - | - |
| Engagement - motivation | Liked NoObesity name | Medical name is good Opinion of name? (2) Prevent from downloading? (0) | Negative / positive connotations | The connotations of the app were important for some of the participants, who felt that a more positive focus on health and behaviour was better than a focus on weight and obesity | Either | Motivation (automatic) |
| Engagement - motivation | Issues with name | Can see why others wouldn't like Make name more positive Negative connotations Opinion of name (0,1) Prevent from downloading (1,2) |  |  |  |  |
| Engagement - motivation | Make app more positive | Positive feedback / encouragement Focus on feelings not measurements |  |  |  |  |
| Engagement - motivation | Issues with notifications | Daily notifications too much Didn't take much notice of notifications Noise of notification frustrating Notifications came at bad times | Notifications | Notifications were seen as helpful reminders to use the app, but parents wanted to be able to decide when was a convenient time to receive them | Either | Capability (psychological) Motivation (automatic) Motivation (reflective) |
| Engagement - motivation | Improve notifications | Prompts / reminders Targeted notifications |  |  |  |  |
| Engagement - motivation | Not enough variety - repetitive | Repetitive (games) Repetitive (goal setting) Simple goals (0) | Novelty / variety | Participants wanted new content to encourage them to keep looking at the app | Positive | Capability (psychological) Motivation (automatic) |
| Engagement - motivation | Not enough variety - limited content | Limited things to do Liked recording progress (0) Boring Nothing new Mostly used to record progress Not realistic (healthy swaps) Looked for suggestions / ideas outside of app Liked healthy eating swaps (0) Flexibility (0 - goal setting) |  |  |  |  |
| Engagement - motivation | Not memorable | Liked recording progress (0) Not memorable (family survey) Forgot to keep up |  |  |  |  |
| Engagement - motivation | More content and more variety of content | More variety More information / education Home workouts Local activities Ways to overcome personal barriers Recipes / meal planning / shopping support Vegetarian / vegan options |  |  |  |  |
| Engagement - motivation | Elements liked in Family Survey | Personalisation (1 - family survey) Inclusion of other healthy behaviours Liked oral health addition | Personalisation | Participants liked the option to put in a family photo and expressed desire to make the app more personal to them | Positive | Capability (psychological) |
| Engagement - motivation | Increased personalisation | Personalisation Separate goals |  |  |  |  |
|  | | | | | | |
| Engagement - usability | Good usability | Easy to use (1 - goal setting) User friendly / easy to use (2) Kid friendly (2) | Ease of use | Most participants felt that the app was easy to use, but some noted that reducing the effort required to use the app would make them more likely to use it | Positive | Capability (psychological) |
| Engagement - usability | Poor usability | User friendly / easy to use (0,1) Kid friendly (0,1) Differences within family |  |  |  |  |
| Engagement - usability | Improve usability | Interface redesign Date of birth rather than age Back button functionality Intro / guide to use |  |  |  |  |
| Engagement - usability | Lack of clarity | Points not clear / need more meaning Unclear how it would work Confused about who it was for | Lack of clarity / guidance | Several participants felt that a lack of clarity hindered their use of the app and suggested incorporating a guide or introduction to clarify its aims and function | Negative | Capability (psychological) Motivation (automatic) |
| Engagement - usability | Lack of guidance | Lack of guidance (usability) Easy to use (0 - goal setting) Guidance (0 - goal setting) No help function |  |  |  |  |
| Engagement - usability | Specific complaints | Specific complaints Technical difficulties Specific complaints (games) Can't change inputted data Inaccuracies | Technical difficulties | Some participants reported issues of the app freezing, getting locked out, or not being able to click on the goals that interfered with their use | Negative | Opportunity (physical) |
| Engagement - usability | Barriers to app use | *Child's age Covid-19 Device* | Unable to use | Children's engagement with the app was limited by their age | Negative | Opportunity (physical) |
|  | | | | | | |
| Perceived impact - motivation | App was motivating | App helped motivate (1) Accountability App increased awareness of behaviour App increased awareness of weight Liked recording progress? (1) Recording progress was motivating? (1) Prompted behaviours | Awareness of behaviour | Goal setting and self-monitoring features reminded participants to pay attention to their current behaviour and whether it aligned with their goals (helped to hold themselves accountable) | Positive | Motivation (automatic) Motivation (reflective) Capability (psychological) |
| Perceived impact - motivation | Liked family aspect | Liked family involvement / doing together | Doing as a family | Participants liked the prompt to consider what was healthy for the family as a whole and do healthy activities together with everyone involved | Positive | Opportunity (social) |
| Perceived impact - motivation | Not motivating | App helped motivate (0) Engagement / use of app (0) Recording progress was motivating (0) | *Descriptive sub-theme (not a factor)* | - | - | - |
|  | | | | | | |
| Perceived impact - self-efficacy | More content and more variety of content | More variety More information / education Home workouts Local activities Ways to overcome personal barriers Recipes / meal planning / shopping support Vegetarian / vegan options | App suggestions (for behaviours) | Healthy eating and activity swaps helped give parents easy ideas for improving their behaviours | Positive | Capability (psychological) |
| Perceived impact - self-efficacy | Suggestions were helpful | App supported self-efficacy? (1) Overcoming barriers helps with self-efficacy Suggestions supported self-efficacy (1) Liked activity suggestions? (1) Liked healthy eating swaps? (1) Liked parent survival guide? (1) Links to other resources were useful Goals helped with planning (1) Liked goal suggestions / found helpful Guidance (1 - goal setting) |  |  |  |  |
| Perceived impact - self-efficacy | Liked goal setting | Flexibility (1) Goals helped with planning Liked goal setting? (2) Simple goals Thinking about barriers was useful | Goal setting support | Some participants noted finding the app's suggestions for goals and prompts to consider barriers helpful for setting goals and making plans | Positive | Capability (psychological) |
| Perceived impact - self-efficacy | Didn't support self-efficacy | App supported self-efficacy (0) Can't overcome some barriers | *Descriptive sub-theme (not a factor)* | - | - | - |
| Perceived impact - self-efficacy | Liked app feedback | Notifications useful / lacking? (1) Feedback useful? (1) Liked points trophies? (1) | Useful feedback | Feedback from the app | Positive | Capability (psychological) Motivation (automatic) Motivation (reflective) |
| Perceived impact - self-efficacy | Liked app feedback | Notifications useful / lacking? (1) Feedback useful? (1) Liked points trophies? (1) | Notifications | Prompts from the app were generally seen as helpful to remind the users to engage with the behaviour | Either | Capability (psychological) Motivation (automatic) Motivation (reflective) |
|  | | | | | | |
| Perceived impact - health behaviours | Perceived behavioural benefits | Ate more healthy (1) More active / outside more (1) Increased water consumption Tried new things | *Descriptive sub-theme (not a factor)* | - | - | - |
| Perceived impact - health behaviours | Didn't improve health behaviour | Didn't help Already doing behaviour More active / outside more (0) Ate more healthy (0) Healthy eating harder than activity | Already doing behaviour | Participants who were already active or eating mostly healthy didn't think the app had an impact on their behaviour | Negative | Motivation (reflective) |
| Perceived impact - health behaviours | Barriers to behaviour | Accessibility Other health conditions | Lack of accessability / affordability | Participants highlighted several barriers relating to accessibility, including the availability of local, affordable activities, safe spaces for children to be active outside, and the cost of healthy food | Negative | Opportunity (physical) |
| Perceived impact - health behaviours | Barriers to behaviour | Child's age Obesity management different for kids vs adults Picky eaters Sibling teasing | Family dynamics | A variety of different family dynamics were mentioned that influenced family's weight-related behaviours, including children's willingness to eat certain foods, their age and ability to do certain activities, and sibling teasing | Either | Capability (physical) Capability (psychological) Opportunity (social) |
| Perceived impact - health behaviours | Barriers to behaviour | Covid-19 | Covid-19 | Covid-19 restrictions limited families options for activities that they could do and affected their motivation to do healthy behaviours | Negative | Opportunity (physical) |
| Perceived impact - health behaviours | Barriers to behaviour | Lack of skills | Lack of skills / ability | While none of the participants reported a lack of skills as a personal barrier, it was mentioned as a potential barrier that could hinder some people in preparing healthy meals | Negative | Capability (physical) Capability (psychological) |
| Perceived impact - health behaviours | Barriers to behaviour | Lack of motivation (convenience)  Lack of accountability Habits / routines | Lack of motivation | A lack of motivation - just "not wanting to" - was often a barrier to making an extra effort to do a healthy behaviour | Negative | Motivation (automatic) Motivation (reflective) |
| Perceived impact - health behaviours | Barriers to behaviour | Lack of time | Lack of time | Finding time to prepare healthy meals or do activities as a family was a barrier | Negative | Opportunity (physical) |
| Perceived impact - health behaviours | Barriers to behaviour | Weather | Bad weather | Temperature, daylight, and weather conditions affected participants' motivation and ability to go outside to be active | Negative | Motivation (automatic) Motivation (reflective) Opportunity (physical) |
|  | | | | | | |
| Perceived impact - communication with HCP | Positive opinions about HCP link | Accountability | Accountability | Some participants mentioned that having a sense of accountability to a HCP who could see their progress would help motivate them to do their goals | Positive | Motivation (reflective) Opportunity (social) |
| Perceived impact - communication with HCP | Positive opinions about HCP link  Concerns with linking with HCP | Comfort discussing with HCP? (2) Communicating via app (1) Want human connection through app Would link with HCP? (1) App data didn't reflect true behaviour Comfort discussing with HCP (0,1) Depends on clinician Sensitive topic Would link with HCP (0,1) Felt that it was inappropriate HCP feedback useful? (0) Wouldn't want to bother HCP | Comfort / trust with HCP | Many participants felt that the benefit of having a linked HCP would depend on how comfortable they felt discussing weight-related issues with them, with one participant noting that they would worry that the recorded progress on the app wouldn't reflect their true behaviour and would give the wrong impression | Positive | Motivation (automatic) Motivation (reflective) Opportunity (social) |
| Perceived impact - communication with HCP | Concerns with linking with HCP | Communicating via app (0) Privacy / security concerns | Privacy / security concerns | One participant mentioned that they would want to understand how personal data was used and protected before sharing anything via the app | Negative | Motivation (reflective) |
| Perceived impact - communication with HCP | Positive opinions about HCP link | Could integrate with other health services / family HCP feedback useful? (1) | Useful feedback (from HCP) | Most participants felt that having the HCP be able to provide feedback based on their family's goals and progress and make suggestions would be helpful | Positive | Capability (psychological) Motivation (automatic) Motivation (reflective) Opportunity (physical) |
