# Supplementary material for: Barriers and facilitators to parents’ engagement with and perceived impact of a childhood obesity app: A mixed-methods study
Source: PLOS Digit Health. 2024 Mar 27;3(3):e0000481. doi: 10.1371/journal.pdig.0000481 (PMC10971669; doi:10.1371/journal.pdig.0000481)
Supplement: S6 Table — (DOCX) [file pdig.0000481.s007.docx]

S6 Table compiles previously published information from [1-4]

| **COM-B** | **Definition [1]** | **TDF** | **Definition [2]** | **Examples (engagement with mHealth) [3,4]** |
| --- | --- | --- | --- | --- |
| Physical capability | Physical skill, strength or stamina | Skills | An ability or proficiency acquired through practice | - App literacy: Technological competency |
| Psychological capability | Knowledge or psychological skills, strength or stamina to engage in the necessary mental processes |  |  |  |
|  |  | Knowledge | An awareness of the existence of something | - App awareness: Knowledge of the existence of health and well-being apps - User guidance: Instructions on how to effectively use the app - Health information: Educational information related to health and well-being aspects - Statistical information: A visual or numerical summary of progress |
|  |  | Memory, attention, and decision processes | The ability to retain information, focus selectively on aspects of the environment and choose between two or more alternatives | - Well-designed reminders: The ability to customize reminders - Less cognitive load: The app is not too time consuming, easy to use, and requires minimal input - Reminders: Preferably customizable notification-type messages |
|  |  | Behavioural regulation | Anything aimed at managing or changing objectively observed or measured actions | - Self-monitoring: The ability of the app to help self-regulation of the target behavior - Established routines: Regularity in using the app - Routines: The ability to support routine or habit formation - Safety netting: Retaining the app for a potential upcoming event in the future - Stepping-stone: App as a first step in the behavior change process |
| Physical opportunity | Opportunity afforded by the environment involving time, resources, locations, cues, physical ‘affordance’ | Environmental context and resources | Any circumstance of a person’s situation or environment that discourages or encourages the development of skills and abilities, independence, social competence and adaptive behaviour | - Availability and accessibility: The ability to use a smartphone anytime anywhere - Low cost: the price of the app - Interactive and positive tone: Encouraging communication style - Personalization to needs: The possibility to use an app that is tailored to a user’s needs - Tailoring: Innovative features, adaptability, and interactive and 2-way communication between the app and user |
| Social opportunity | Opportunity afforded by interpersonal influences, social cues and cultural norms that influence the way that we think about things, e.g. the words and concepts that make up our language | Social influences | Those interpersonal processes that can cause individuals to change their thoughts, feelings, or behaviours | - Peer support: Including social interaction with users with similar needs within the app or within their community; a choice to connect to social media platforms, competitions, and challenges with others or with themselves - Social support (practical): Possibility to contact health professionals and practitioners within the app - Recommendations: Suggestions received from other users - Health practitioner support: Possibility to get in touch with health professionals and practitioners within the app - Community networking: Social interaction with users with similar needs within the app or within their community - Social media: A choice to connect to social media platforms - Social competition: Competitive nature of the app with others or with themselves - Personification of the app: Applying human attributes to the app |
| Reflective motivation | Reflective processes involving plans (self- conscious intentions) and evaluations (beliefs about what is good and bad) | Social / professional role and identity | A coherent set of behaviours and displayed personal qualities of an individual in a social or work setting |  |
|  |  | Beliefs about capabilities | Acceptance of the truth, reality or validity about an ability, talent or facility that a person can put to constructive use | - Self-confidence: Perceived capability to change one’s behavior using an app |
|  |  | Optimism | The confidence that things will happen for the best or that desired goals will be attained |  |
|  |  | Beliefs about consequences | Acceptance of the truth, reality, or validity about outcomes of a behaviour in a given situation | - Perceived utility of the app: Discrepancy of what the users are looking for and what the app offers - Commitment: The level of commitment while engaging with an app to change the behavior and achieve set goals |
|  |  | Intentions | A conscious decision to perform a behaviour or a resolve to act in a certain way |  |
|  |  | Goals | Mental representations of outcomes or end states that an individual wants to achieve | - Goal setting: Establishing what the user would like to accomplish - Action planning: Establishing how the user would like to achieve set goals |
| Automatic motivation | Automatic processes involving emotional reactions, desires (wants and needs), impulses, inhibitions, drive states and reflex responses | Reinforcement | Increasing the probability of a response by arranging a dependent relationship, or contingency, between the response and a given stimulus | - Feedback: Feedback regarding the user’s performance - Rewards: Tangible (eg, objects and discounts) and intangible (eg, badges and certificates) rewards in response to the user’s effort and gamification elements - Encouragement: Additional ways to provide reinforcement (eg, encouraging messages) |
|  |  | Emotion | A complex reaction pattern, involving experiential, behavioural, and physiological elements, by which the individual attempts to deal with a personally significant matter or event | - Curiosity: Desire to acquire knowledge and skills to use a behavior change tool - Positive emotions: Triggered by the included user guidance, statistical information, additional health information, embedded professional support, community networking possibilities, tracking features, and rewards - Negative emotions: Triggered by lack of user guidance, invasive push notifications, cognitive overload, and unrevealed in-app costs - Mixed emotions: Triggered by reminders |

**S6 Table References**

1. Michie S, Atkins L, West R. The Behaviour Change Wheel: A Guide to Designing Interventions. Silverback Publishing; 2014.

2. Atkins L, Francis J, Islam R, O’Connor D, Patey A, Ivers N, et al. A guide to using the Theoretical Domains Framework of behaviour change to investigate implementation problems. Implement Sci. 2017;12: 1–18.

3. Szinay D, Jones A, Chadborn T, Brown J, Naughton F. Influences on the Uptake of and Engagement With Health and Well-Being Smartphone Apps: Systematic Review. J Med Internet Res. 2020;22: e17572.

4. Szinay D, Perski O, Jones A, Chadborn T, Brown J, Naughton F. Perceptions of Factors Influencing Engagement With Health and Well-being Apps in the United Kingdom: Qualitative Interview Study. JMIR Mhealth Uhealth. 2021;9: e29098.
