# Supplementary material for: Barriers and facilitators to parents’ engagement with and perceived impact of a childhood obesity app: A mixed-methods study
Source: PLOS Digit Health. 2024 Mar 27;3(3):e0000481. doi: 10.1371/journal.pdig.0000481 (PMC10971669; doi:10.1371/journal.pdig.0000481)
Supplement: Attachment — Submitted filename: Response to Reviewers_PDIG-D-23-00411.docx [file pdig.0000481.s008.docx]

| PLOS Digital Health |  |  |
| --- | --- | --- |

06.12.2023

Dear Haleh Ayatollahi,

Thank you for your consideration of our manuscript “Barriers and facilitators to engagement with and impact of a childhood obesity app: a mixed-methods study” for publication in PLOS Digital Health. We have made amendments to the manuscript reflecting the valuable review comments forwarded and feel the paper has been well enhanced to be re-presented for publication. Please let us know if there is anything we can further do to clarify or extend our paper.

With kind regards,

Edward Meinert

Enclosures:

1. Chart mapping peer review feedback

| **ID** | **Reviewer** | **Reviewer Feedback** | **Author response** | **Action taken** |
| --- | --- | --- | --- | --- |
| 1 | Reviewer 1 | Please follow data sharing and availability rules for PLOS Digital Health. It is not permissible to put the corresponding author as the primary point of contact for data sharing----there are ways around institutional restrictions. | We thank Reviewer 1 for their time and consideration in the review of our manuscript. We have amended the data availability statement and made the anonymized dataset available in OSF. | Dataset deposited in OSF, data availability statement amended |
| 2 | Reviewer 2 | Regarding the checklist, we aim to provide clarity to the authors and acknowledge their efforts in advance. | We thank Reviewer 2 for their time and consideration in the review of our manuscript. The raised issues have been addressed in view of the peer review feedback provided. | N/A |
| 3 | Reviewer 2 | TITLE and ABSTRACT Clarification is needed on how units were allocated to interventions. | Thank you for highlighting that this was not clear; we have amended the abstract to clarify that all participants were allocated to the intervention (it was a single-arm study). | Abstract amended |
| 4 | Reviewer 2 | Under the section "Information on target population or study sample": The article lacks information on the target population. As it deals with a diverse group, specifically the pediatric population, details about age need to be provided as it represents a distinct patient group. Additionally, the article doesn't cover location or ethnicity. It's suggested to include information regarding the age range of children and parents since the study measures facilitators. Considering the variability in age among participants, it's crucial to account for this variable. | The ethnicity and age range of participants was already included in Table 4 (Participant characteristics), but we thank the reviewer for pointing out additional demographic data that was missing. We have added further demographic data about the study sample to Table 4. Additionally, we have clarified the target population in the ‘Sample and recruitment’ section of the methods and added the lack of data about children’s ages to the limitations as an important data point to collect in future studies. It was not collected in this study as the main participants were parents; however, we observed in the thematic analysis that children’s age was an important factor influencing engagement and perceived impact, and should be examined further in future evaluations. | Added demographic data to Table 4, amended ‘Sample and recruitment’ and Strengths and limitations’ |
| 5 | Reviewer 2 | A structured abstract is recommended: It includes a structured summary that meets the description. | We drafted the abstract based on the PLOS Digital Health guidance, which says that “While the Abstract is conceptually divided into three sections (Background, Methodology/Principal Findings, and Conclusions/Significance), do not apply these distinct headings to the Abstract within the article file.” | N/A |
| 6 | Reviewer 2 | Scientific background and rationale The provided explanation appears rational for the objective of the work presented. The primary objectives of the work are also clearly stated. | N/A | N/A |
| 7 | Reviewer 2 | Eligibility criteria for participants The primary concern observed pertains to the external validity of the work. The sampling method used is convenience sampling rather than stratified. The target population is widely dispersed. As the ultimate goal is to generalize results in the pediatric population, this study includes only 35 subjects without describing the characteristics of the intervened pediatric population. Furthermore, with a wide age range (30 years) within the subject group, randomization could better generalize most of these factors. However, in this study, they are included through diffusion and participation, leading to significant selection bias. Inclusion of voluntary participants implies self-selection bias, limiting generalization to only those willing to participate, thus not reflecting the broader population. | We agree with the reviewer that the sample is the main limitation of the study, which we have discussed in the limitations section. However, the purpose of the study was to examine factors influencing real world use of the intervention – beyond a study, users’ engagement with the intervention will be voluntary. Although randomized controlled trials are valuable for assessing efficacy against a comparator, that was not the primary purpose of this particular study, although it will be an important subsequent investigation. We have clarified this limitation in the manuscript. | Limitations section amended |
| 8 | Reviewer 2 | Method of recruitment Once again, the text doesn't mention a randomized method for registration and participant selection. Self-selection bias is noteworthy, where respondents with specific characteristics are more inclined to participate, potentially skewing the results. Respondents' inclinations towards certain topics lead to unintended biases affecting the study's rationality. Voluntary response bias further limits representation to only those with strong opinions, disregarding others. Ethical constraints prevented an assessment of this bias. Selecting parents from a nearby community through random sampling would have been advisable to achieve a more representative sample. | This study, which focused on factors influencing real-world engagement and perceived impact, did not use a randomized design or a comparator for the overall study sample. Stratified random sampling was used to select the initial sample of participants invited for interview; this has been clarified in the text. We appreciate the reviewer’s suggestion for an alternate means of obtaining a sample; this will be considered for future studies, although we would argue that this geographic restriction would also limit generalizability. However, a larger scale study could potentially sample a set of communities with different socio-economic characteristics to achieve a more generalizable sample. | The limitations section has been revised to incorporate these points.  The sample characteristics section has been amended. |
| 9 | Reviewer 2 | Details of interventions The article specifies a series of questionnaires, which are appended and considered relevant. The authors adequately address these aspects. | N/A | N/A |
| 10 | Reviewer 2 | Ethical considerations The article shows records of the ethics committee R62092/RE001. | N/A | N/A |
| 11 | Reviewer 2 | Data collection instruments and technologies A comprehensive description is provided, considered appropriate. | N/A | N/A |
| 12 | Reviewer 2 | Specific objectives and hypotheses Clear specific objectives are outlined. However, the achievability of the third objective seems questionable given the study's design. Omitting this objective is suggested due to the speculative nature of both the results and the study's context and methodology. | We appreciate the reviewer’s perspective and have removed the third objective as suggested. | Introduction amended |
| 13 | Reviewer 2 | Summary of results and statistical design For each primary and secondary outcome, a summary of results for each estimation study condition, along with the estimated effect size and confidence intervals indicating precision, were adequately presented. The statistical design for the proposed model is considered appropriate. | N/A | N/A |
| 14 | Reviewer 2 | Integration with prior work and implications In the discussion, it would be beneficial to compare these results with previous studies, citing another intervention in a similar context and its identified domains and impacts. | We have identified a couple of similar studies, although there are few that conduct the same type of analysis of barriers and facilitators with digital health engagement within the context of childhood obesity; this has been added to the discussion. | Discussion amended |
| 15 | Reviewer 2 | Final Considerations: Acknowledging this as a commendable piece of work, restructuring known weaknesses is necessary. While redoing the work may not be feasible, revising objectives and methods to better align with the proposed objectives is crucial. Mere theorization cannot suffice as a response to an objective. Reviewing the text content with the suggested adjustments and addressing the comments upon resubmission is recommended. | We thank the reviewer for their thorough feedback, thoughts, and suggestions for improvement. We have addressed the comments as described above. | N/A |
| 16 | Reviewer 3 | It is an interesting manuscript analyzing barriers and facilitators to engagement with an app designed to impact on children obesity. | We thank Reviewer 3 for their time and consideration in the review of our manuscript. The raised issues have been addressed in view of the peer review feedback provided. | N/A |
| 17 | Reviewer 3 | The authors report that only 16% of the parents that gave their consentient participated in the study mainly because they didn’t meet the inclusion criteria. However, they do not suggest any strategy that could be applicable in future studies to increase the sample. | The participation rate was not related to the eligibility criteria; we have clarified the flow diagram to better explain this. We have also added recommendations to the discussion for how sample size could be improved in future research. | Discussion and Figure 3 amended |
| 18 | Reviewer 3 | The authors designed the study including the participation of three important groups, parents, HCP and HEE. One of the limitations of the study is that no HCP participated. The authors do not discus neither clearly indicate the weight of this limitation in the lack of engagement (only 34% of the sample used the app at the end of the study). The HCPs could have played an important role in the parents' commitment to use the app and so this could have been a very important factor in the results. | We agree that this is a limitation of the study and an area where future research is needed. We have added a separate paragraph to the discussion to discuss this point. | Discussion amended |
| 19 | Reviewer 3 | Even though the authors included appropriate references they do not give evidence of the originality of the study, because they do not compare their approach or results with other studies | The results of the study are compared to many of evaluations of factors influencing engagement with digital health in the discussion. These are not necessarily specific to childhood obesity, as that would limit the assessment of the generalizability of barriers and facilitators identified and the limited number of studies that conduct similar evaluations in that particular clinical case. We have added a paragraph explaining this in the discussion. | Discussion amended |
| 20 | Reviewer 4 | This well-written manuscript presents an evaluation of a weight management intervention for children that is used in the family context. It uses data collected via a mixed methods approach to study barriers and facilitators to intervention use and behaviour change. Grounding the evaluation in multiple theories and frameworks and providing helpful visualisations of the qualitative findings are clear strengths of the manuscript. There are a few open questions that might need to be addressed prior to publication: | We thank Reviewer 4 for their time and consideration in the review of our manuscript. The raised issues have been addressed in view of the peer review feedback provided. | N/A |
| 21 | Reviewer 4 | 1) Why was engagement with app assessed via first and last login and not via the number of logins or use of intervention features? | The system only collected minimal app usage data, that unfortunately did not included number of logins of use of intervention features. We have clarified this point in the limitations and outlined how it will be addressed in future research. | Limitations amended |
| 22 | Reviewer 4 | 2) To further understanding inclusivity of the intervention it might be insightful - if the data is available - to compare the final sample to the enrolled sample in terms of demographics. | We agree that this would have been a valuable analysis; unfortunately, consent and demographic surveys were not conducted at the same time, and only 61 of the participants who originally consented provided demographic data, so it was not possible for us to compare the final and enrolled samples in terms of demographics. We have added a sentence addressing this point to the limitations and highlighting the benefit of capturing demographic data immediately after consent to enable such comparisons in future research. | Limitations amended |
| 23 | Reviewer 4 | 3) The recommendations provided in Table 6 are helpful, but not necessarily rooted in the presented data (e.g., many recommendations focus on HCPs which could not be studied). Please include references to the literature for all recommendations that were derived from the literature instead of from the data at hand. | As suggested, we have added citations for elements of the recommendations that were derived from the literature. | Table 6 amended |
| 24 | Reviewer 4 | 4) It's a shame that HCPs did not take part in the evaluation, and that they were generally not contacted by the app users. What are the reasons for HCPs not being more involved in the evaluation, and how could exchange between HCPs and users be promoted? This might be especially crucial since there is first evidence that including personal contact in apps might promote engagement and effectiveness compared to digital-only services, and that in person interventions are generally more effective than digital weight loss interventions: Dombrowski, S., Whitcomb, E., Olthuis, J., Witherspoon, R., & Hebert, J. (2021). Syetemactire review of RCTs comparing face-to-face vs remote behaviour change interventions for weight management. In 35th Conference of the European Health Psychology Society. Mamalaki, E., Poulimeneas, D., Tsiampalis, T., Kouvari, M., Karipidou, M., Bathrellou, E., ... & Yannakoulia, M. (2022). The effectiveness of technology‐based interventions for weight loss maintenance: a systematic review of randomized controlled trials with meta‐analysis. Obesity Reviews, 23(9), e13483. | We agree with the reviewer’s comment and would have liked to be able to investigate this; unfortunately, we were unable to collect data about HCPs reasons for non-participation or drop-out. We have added a paragraph to the discussion addressing this point. | Discussion amended |
| 25 | Reviewer 5 | The paper aimed to explore barriers and facilitators to engagement with a family-focused app for childhood obesity prevention and its perceived impact on motivation, self-efficacy, and behaviour. The topic is relevant and up-to-date, and the findings highlighted the importance of examining how a digital health intervention could be designed and implemented. Although the method session, especially the sampling and recruitment needs some clarification, the study is robust, well-written, and provides important insights for future interventions and scaling-up. Some minor comments are suggested below: | We thank Reviewer 5 for their time and consideration in the review of our manuscript. The raised issues have been addressed in view of the peer review feedback provided. | N/A |
| 26 | Reviewer 5 | Title: Since the paper assessed the perceived impact on motivation, self-efficacy, and behaviour, not the intervention impact on childhood obesity per se, it is suggested to include “perceived impact” instead of only “impact” in the title for clarification.  How it is described may lead the reader to think the paper evaluated the impact of the app intervention on childhood obesity. | We appreciate the reviewer’s argument and have amended the title as suggested. | Changed “impact” to “perceived impact” in title |
| 27 | Reviewer 5 | On page 6, session “Intervention”, the authors stated that: “The target audience included families wanting behavioural support (regardless of weight)”. Please, explain a little more about how the app is routinely used. How are the users targeted, and how can they reach support with the app? Is the app publicly available? Is there any publicity on the app so that families who want behavioural support know how to seek it? | At the time of the study, the app was publicly available on the Apple App Store and Google Play and advertised on Health Education England’s website. As the app is in the process of being redesigned, it is not currently available for download, but will be again once this has been completed. We have included this information in the Intervention section as suggested. | Intervention section amended |
| 28 | Reviewer 5 | On page 7, session “Sample and recruitment”, what was the justification for the exclusion of “children and vulnerable adults”? What was defined as a vulnerable population? | Thank you for highlighting the lack of clarity; we have clarified the exclusion criteria in the Sample and recruitment section. | Sample and recruitment section amended |
| 29 | Reviewer 5 | On page 8 “Stratified random sampling (based on gender, ethnicity, and income) was used to select a subset of parents for semi-structured interviews (SSIs)”. Was it out of the 61 parents who completed the demographic survey? How was the final number of 15 interview parents reached? What were the proportions of each sampling strata? | We have added the demographics of the interviewed sample to Table 4 to improve clarity and amended the Sample and recruitment and sample characteristics sections to specify how the sample was selected and who it included in the analysis. | Sample and recruitment and sample characteristics sections amended, Table 4 amended |
| 30 | Reviewer 5 | On page 12, session “Sample characteristics”. It is important to clarify in the protocol people who were lost during the follow-up, and those, who were excluded due to incomplete data (but still finish the study). If participants could complete the study regardless of if they filled out the initial forms, then, they were not lost for follow-up. Please, review the flow diagram. | We thank the reviewer for raising this point; we have revised the flow diagram to clarify at what stages participants were lost from follow-up or excluded. | Flow diagram revised |
| 31 | Reviewer 5 | On page 12, please clarify if a participant would still use the app after consent, but not complete any of the questionnaires. How was this controlled? Is there any information about the initial sample of parents (225)?  The sample is a bit confusing. Please, clarify it. It would be interesting to see some initial characteristics for the total population (225) to see if they differ from people who were not lost for follow-up. | Thank you for identifying this absence in the text; we have explained that participants who did not complete the study may still have been using the app, as it was publicly available and not restricted.  As discussed in the response to ID 22, we agree that it would have been interesting to compare characteristics of the recruited sample with the final sample; unfortunately, this was not possible as consent and demographic surveys were not conducted at the same time, and only 61 of the participants who originally consented provided demographic data. We have added a sentence addressing this point to the limitations and highlighting the benefit of capturing demographic data immediately after consent to enable such comparisons in future research. | Sample characteristics and limitations sections amended |
| 32 | Reviewer 5 | Page 14 – “only half of final survey respondents (19/35, 54%) reported still using the app and only a third had recently logged in by the end of the study (9/26, 34%)”. It is not clear what this “a third” means (9/26). Where do these 26 people come from, if there were 35 interviewees, and 26 is not a third of 35? | This related to the participants who provided app use data; this has been clarified in the text. | Engagement with the intervention section amended |
| 33 | Reviewer 5 | Page 14 – “On average, there were 5.1 months between account setup and most recent login (95% CI: 3.9-6.3, range: 0.0-10.4 months).” How is the maximum time 10.4 months if the intervention duration was 6 months, and only individuals with no previous use of the app were recruited? | App use data was collected after the end of the 6-month intervention period (in June 2021). This is why some of the participants had engagement periods longer than 6 months. We have added the date of the app use data collection to the Procedure and Engagement with the intervention sections to clarify this point. | Procedure and Engagement with the intervention sections amended |
| 34 | Reviewer 5 | Page 17: “Perceived impact on health behaviours was mixed and often conflicting; most participants reported some progress on their goals, but many said that the app did not have an impact on their behaviour.” The paper needs to make it clear how is the app designed to interact with parents on the aim of behaviour changes to overcome childhood obesity in the family context. The way it is presented, it is not clear how these goals and changes reported by parents are aligned with the main app goal. It would be helpful to describe some of the goals, and how they are applied in the family context, not the individual. | We thank the reviewer for pointing out this potential area of confusion; we have added a couple of example goals that the app suggests to parents. | Intervention section amended |
| 35 | Reviewer 5 | Page 27: Limitations. Please, address the limitations regarding the exclusion of vulnerable people, the method of recruitment (social media advertising), and how this could introduce bias in the sample. | In line with this feedback and comments from other reviews (IDs 7 and 8), we have added a discussion of these points into the limitations section. | Limitations section amended |
