# Supplementary material for: Barriers and facilitators to parents’ engagement with and perceived impact of a childhood obesity app: A mixed-methods study
Source: PLOS Digit Health. 2024 Mar 27;3(3):e0000481. doi: 10.1371/journal.pdig.0000481 (PMC10971669; doi:10.1371/journal.pdig.0000481)
Supplement: Attachment — Submitted filename: Response-to-Reviewers-v2.docx [file pdig.0000481.s009.docx]

| PLOS Digital Health |  |  |
| --- | --- | --- |

12.01.2024

Dear Professor Ayatollahi,

Thank you for your consideration of our manuscript “Barriers and facilitators to engagement with and perceived impact of a childhood obesity app: a mixed-methods study” for publication in PLOS Digital Health. We have made amendments to the manuscript reflecting the valuable review comments forwarded and feel the paper has been well enhanced to be re-presented for publication. Please let us know if there is anything we can further do to clarify or extend our paper.

With kind regards,

Edward Meinert

Enclosures:

1. Chart mapping peer review feedback

| **ID** | **Reviewer** | **Reviewer Feedback** | **Author response** |
| --- | --- | --- | --- |
| 1 | Reviewer 2 | The main concern observed persists, which relates to the external validity of the work. The sampling method used is convenience sampling rather than stratified. The target population is widely dispersed. Since the ultimate goal is to generalize the results to the pediatric population, this study includes a small number of subjects without describing the characteristics of the intervened pediatric population, which is an important factor. Upon author review, it is commented once again that the corrections were only made in a theoretical manner. I believe that due to the type of sampling, we cannot generalize the results as stated in the primary objective. It is suggested to increase the sample size or modify the title to be more specific to the scope of the external validity of the work. No corrections were made regarding this point, so I suggest once again modifying the scope of the work or specifying objectives that may be more achievable with the proposed design. | We appreciate the reviewer’s point and agree that their concern about the external validity of the work is an extremely important consideration; as such, we are keen to fully address this point. The reviewer is concerned that, due to the sample size and demographics of our study, the results may not be generalizable to the pediatric population and raises the point that we did not capture pediatric characteristics. We will respond to these concerns as two points, to ensure both are thoroughly addressed.  First, we agree with the reviewer that due to the sample size and demographics, we could not claim statistical generalizability of the results to the whole pediatric population. The aim of our study was not to predict on a population-level how users would engage with or benefit from the intervention but to generate, through in-depth analysis of participants’ experiences, key and common factors that influence engagement with and perceived impact of a digital health app. To achieve this aim, we conducted a primarily qualitative analysis, which is well-equipped to explore such experiences in detail. Delving in-depth into the experiences of a small number of participants is a strength of qualitative research, but it does preclude the pursuit of the type of statistical generalizability as the reviewer has described. Instead, other types of generalizability can be sought for qualitative research ([Smith, 2018](https://doi.org/10.1080/2159676X.2017.1393221); [Varpio et al. 2020)](https://asmepublications.onlinelibrary.wiley.com/doi/pdf/10.1111/medu.14348?casa_token=9GCB_L77EqoAAAAA%3AvYemwncSK-vLPfz_k-c04vJpKBpt-quGBPjSbIZLArXD7mxx89UthfzxaFahx_-M7t6rDhr3PktlSExu). For example, in our study, we were more interested in the generalizability (or transferability) of the factors identified as associated with engagement and perceived impact, as this has the potential to help shape digital intervention design in both the specific context of childhood obesity and in other health contexts. The alignment of our findings with previous studies in other areas of digital health does suggest some degree of generalizability of these outcomes, as we explored in the discussion. We appreciate the reviewer highlighting the ambiguity of this point and we have amended our phrasing of the study aim to better clarify this point.  Second, as mentioned in our previous response, we focused on the characteristics of the parents, as they were the users primarily engaging with the app. However, we agree that the reviewer is correct to point this out as a limitation, as our analysis identified that factors relating to the children’s characteristics (such as age) could influence engagement and perceived impact. We have incorporated this point into our discussion as a limitation and element to consider in our future research. |
| 2 | Reviewer 3 | It's great to hear that the authors addressed the questions and comments effectively, incorporating changes into the manuscript. Although the sample size and the absence of healthcare professionals (HCPs) in the study are acknowledged as limitations, the reviewer recognizes the study's importance in providing valuable insights. The focus on factors like ethnicity, income, education, and familiar environment for understanding the impact of apps on weight loss in children with obesity is highlighted as a key contribution. The reviewer believes that the substantial changes made by the authors meet the standards for publication in Plos Digital Health | We thank the reviewer for the time and effort they have provided to review and suggest improvements for this paper, and we are glad that they feel their comments have been sufficiently addressed. |
| 3 | Reviewer 4 | (No Response) | N/A |
| 4 | Reviewer 5 | The authors fully addressed the reviewers' comments, clarifying the questions and/or changing the manuscript. Most comments were included in the new version, which enhanced the overall quality of the manuscript and is now in alignment with the points raised by the reviewers. Considering the relevance and design of the study, the potential to contribute to the field, and the overall quality of the paper after this revision, I am recommending acceptance. | We thank the reviewer for the time and effort they have provided to review and suggest improvements for this paper, and we are glad that they feel their comments have been sufficiently addressed. |
