# Supplementary material for: Barriers and facilitators to parents’ engagement with and perceived impact of a childhood obesity app: A mixed-methods study
Source: PLOS Digit Health. 2024 Mar 27;3(3):e0000481. doi: 10.1371/journal.pdig.0000481 (PMC10971669; doi:10.1371/journal.pdig.0000481)
Supplement: Attachment — Submitted filename: Response-to-Reviewers-v3.docx [file pdig.0000481.s010.docx]

| PLOS Digital Health |  |  |
| --- | --- | --- |

23.01.2024

Dear Professor Ayatollahi,

Thank you for your consideration of our manuscript “Barriers and facilitators to engagement with and perceived impact of a childhood obesity app: a mixed-methods study” for publication in PLOS Digital Health. We have made amendments to the manuscript reflecting the valuable review comments forwarded and feel the paper has been well enhanced to be re-presented for publication. Please let us know if there is anything we can further do to clarify or extend our paper.

With kind regards,

Edward Meinert

Enclosures:

1. Chart mapping peer review feedback

| **ID** | **Reviewer** | **Reviewer Feedback** | **Author response** |
| --- | --- | --- | --- |
| 1 | Reviewer 2 | Dear author, we find ourselves in the same situation once again. The comments regarding statistics and models are clear and understood, and I agree with the methodology. However, I believe that clarity in the title is of utmost importance in a study, as one of the fundamental principles of research is simply the alignment of the title with the objectives. The responses I am requesting are simply related to the modification of the title based on the actual objectives you are presenting. To illustrate my point, let me provide an example: The current title reads: Title: Barriers and facilitators to engagement with and perceived impact of a childhood obesity app: a mixed-methods study However, the objectives you present are different, as follows: The purpose of this study was to gain insight into why why people engaged and disengaged with an app for childhood obesity, how it influenced their behaviour, and how this could be improved. Specifically, it aimed to 1) generate barriers and facilitators that influence users’ engagement with the app and 2) to evaluate its impact on perceived motivation, self-efficacy, weight-related health behaviours, and communication between healthcare professionals (HCPs) and families However, in your responses, you use these objectives: "To describe participants' experiences, key factors, and commonalities influencing engagement and perceived impact of a digital health app." This does not align with your general title, as the title suggests that we need to examine how people, including children, engage with the app. Following this logic, it assumes that children are not considered individuals in this analysis. This raises an ethical issue that needs to be addressed. I suggest changing your objectives as follows: 1.      Analyze how some parents engage and disengage with a childhood obesity app, and how it influences their behavior and potential improvements in app usage. 2.      Evaluate the impact on parents in terms of perceived motivation, self-efficacy, weight-related behaviors, and communication with healthcare professionals. Additionally, you mention: "For example, in our study, we were more interested in the generalization (or transferability) of the factors identified as associated with participation and perceived impact, as this has the potential to shape the design of digital interventions in the specific context of childhood obesity and other health contexts." However, we cannot discuss a health context without considering the subjects of the study, as these factors always influence the perception of the phenomenon. For example, I cannot assess the quality of life perception in cancer patients without knowing the cancer stage, the patient's age, and the histological type of cancer, as these factors are crucial in interventions. In conclusion, the requested changes were not made, and the responses remain theoretical. The main issue lies in the title and the objectives, which are not clearly aligned with what is presented in the responses. In future instances, I urge you to be more specific in your objectives, as they determine the methodology to be followed. The title and objectives must be specific to avoid confusion. I appreciate your work and understanding. | Dear reviewer; we appreciate the time you have taken to clarify your feedback and ensure our paper is meeting the expected high and rigorous standard of journal publication. We have amended the title and objectives as you have suggested: we have clarified the focus on parent perspectives by adding ‘parents’ to the title and objectives. Additionally, we have incorporated your other phrasing suggestions for the objectives into the sentences outlining the purpose of the study and the specific objectives. We did not intend to imply that children are not considered individuals; they are an important potential user group of the app that will be included as participants in future studies. We appreciate the reviewer highlighting how the ambiguity of the objectives could make such an implication, because children were not included as participants in this Phase 1 evaluation.  We agree with the reviewer that the specific patient and other characteristics of a particular health context are vitally important considerations for the development of an intervention for that health context. We emphasise in our conclusions that “developers must identify whether these suggested intervention strategies align with engagement barriers in their particular context”. Based on your feedback, we have added to this sentence to make clear that engagement barriers can relate to the patients’ characteristics and other factors. |
