# Supplementary material for: Barriers and facilitators to parents’ engagement with and perceived impact of a childhood obesity app: A mixed-methods study
Source: PLOS Digit Health. 2024 Mar 27;3(3):e0000481. doi: 10.1371/journal.pdig.0000481 (PMC10971669; doi:10.1371/journal.pdig.0000481)
Supplement: Attachment — Submitted filename: Response-to-Reviewers-v4.docx [file pdig.0000481.s011.docx]

| PLOS Digital Health |  |  |
| --- | --- | --- |

08.02.2024

Dear Professor Ayatollahi,

Thank you for your consideration of our manuscript “Barriers and facilitators to engagement with and perceived impact of a childhood obesity app: a mixed-methods study” for publication in PLOS Digital Health. We have made amendments to the manuscript reflecting the valuable review comments forwarded and feel the paper has been well enhanced to be re-presented for publication. Please let us know if there is anything we can further do to clarify or extend our paper.

With kind regards,

Edward Meinert

Enclosures:

1. Chart mapping peer review feedback

| **ID** | **Reviewer** | **Reviewer Feedback** | **Author response** |
| --- | --- | --- | --- |
| 1 | Editor | 1. Please review your reference list to ensure that it is complete and correct. If you have cited papers that have been retracted, please include the rationale for doing so in the manuscript text, or remove these references and replace them with relevant current references. Any changes to the reference list should be mentioned in the rebuttal letter that accompanies your revised manuscript. If you need to cite a retracted article, indicate the article's retracted status in the References list and also include a citation and full reference for the retraction notice. | Regarding the first point about references, we have reviewed all of the included journal article references and no retractions were identified. Since the original submission of the manuscript, no references have been removed and the following references have added:   - Kouvari M, Karipidou M, Tsiampalis T, Mamalaki E, Poulimeneas D, Bathrellou E, et al. Digital Health Interventions for Weight Management in Children and Adolescents: Systematic Review and Meta-analysis. J Med Internet Res. 2022;24: e30675. - González OD-J, Tugault-Lafleur CN, Jean Buckler E, Hamilton J, Ho J, Buchholz A, et al. The Aim2Be mHealth Intervention for Children With Overweight or Obesity and Their Parents: Person-Centered Analyses to Uncover Digital Phenotypes. J Med Internet Res. 2022;24. doi:10.2196/35285 - Tripicchio GL, Kay M, Herring S, Cos T, Bresnahan C, Gartner D, et al. Development and Preliminary Feasibility of iByte4Health: A Mobile Health (mHealth) Pediatric Obesity Prevention Intervention to Engage Parents with Low-Income of Children 2–9 Years. Nutrients. 2021;13. doi:10.3390/nu13124240 - Cornet VP, Daley C, Bolchini D, Toscos T, Mirro MJ, Holden RJ. Patient-centered Design Grounded in User and Clinical Realities: Towards Valid Digital Health. Proceedings of the International Symposium on Human Factors and Ergonomics in Health Care. 2019 [cited 6 Dec 2023]. doi:10.1177/2327857919081023 - Lipschitz JM, Pike CK, Hogan TP, Murphy SA, Burdick KE. The Engagement Problem: a Review of Engagement with Digital Mental Health Interventions and Recommendations for a Path Forward. Current Treatment Options in Psychiatry. 2023;10: 119–135. - Wild CEK, Egli V, Rawiri NT, Willing EJ, Hofman PL, Anderson YC. “It’s more personal if you can have that contact with a person”: Qualitative study of health information preferences of parents and caregivers of children with obesity in New Zealand. Health Soc Care Community. 2022;30. doi:10.1111/hsc.13756   Upon review, three duplicate references were identified and corrected in the most recent manuscript upload:   - Dounavi K, Tsoumani O. Mobile Health Applications in Weight Management: A Systematic Literature Review. Am J Prev Med. 2019;56. doi:10.1016/j.amepre.2018.12.005 was originally included as reference 27 and 38 - Michie S, Atkins L, West R. The Behaviour Change Wheel: A Guide to Designing Interventions. Silverback Publishing; 2014. Was originally included as reference 53 and 75 - Milne-Ives M, Homer S, Andrade J, Meinert E. Associations between Behaviour Change Techniques and engagement with mobile health apps: A systematic review.   The last citation was under review during the initial submission but has since been published, so the reference has also been updated. We have included as an attachment an updated copy of the manuscript with these references highlighted (yellow for additions, blue for amended duplicates). |
| 2 | Reviewer 2 | All comments have been addressed | We thank the reviewer for their time providing their valuable feedback for our paper and their confirmation that their comments have been satisfactorily addressed. We have included responses to each of the final points raised by the reviewer below:   1. ***Regarding collecting data about children’s characteristics*:** As we have said in the first and second rounds of review, we agree that including the general characteristics of the children in the study would have been a useful addition. Unfortunately, the study is now closed and it is not possible to conduct further data collection at this stage. As mentioned in our previous responses, we have added this as a limitation in the Strengths, limitations, and future research section and plan to collect this data in future evaluations. 2. ***Regarding parents' level of education*:** We have already included this data in the paper; data on parents’ level of education is already included in Table 4, broken down by the total sample and the subset of interviewed participants 3. ***Regarding application usage time*:** We have already included a summary of the app usage data that was captured by the NoObesity system in the paper (Section: Engagement with the intervention (adoption and use)). Unfortunately, the app did not record total usage time; the available data was limited to account creation date/time and most recent login date/time. We agree with the reviewer that more detailed data about app usage time would have been valuable, but we did not develop the app being evaluated and it was not designed to capture this data. We have already discussed this limitation in the Strengths, limitations, and future research section. However, we are in the process of redesigning the app based on this study and preparing another evaluation; this is an issue that will be addressed in the app’s re-development. |
| 3 | Reviewer 2 | I am pleased to see that you have made modifications to the text. I believe that delineating the work allows for a better understanding of it. I consider it important to address a couple of necessary issues prior to publication, for which I suggest communicating with the participants:   - Describe the general characteristics of the children under parental care. - Provide information about the parents' level of education. - Share the average usage time of the application.   All of the above is necessary to provide us with a better context of the situation. While I do believe the article has potential, I recommend making these changes before considering it suitable for publication. |  |
